# Enterovirus 71 Neutralizing Antibodies Seroepidemiological Research among Children in Guangzhou, China between 2014 and 2015: A Cross-Sectional Study

**DOI:** 10.3390/ijerph14030319

**Published:** 2017-03-20

**Authors:** Dingmei Zhang, Yan Chen, Xiashi Chen, Zhenjian He, Xun Zhu, Yuantao Hao

**Affiliations:** 1 School of Public Health, Sun Yat-sen University, 74 Zhongshan Road II, Guangzhou 510080, China; zhdingm@mail.sysu.edu.cn (D.Z.); chenxsh25@mail.sysu.edu.cn (X.C.); hezhenj3@mail.sysu.edu.cn (Z.H.); 2 Medical College of Shaoguan University, No.1 Xinhuanan Road, Shaoguan 512000, China; 13826318190@163.com; 3 Zhongshan School of Medicine, Sun Yat-sen University, 74 Zhongshan Road II, Guangzhou 510080, China; zhuxun8@mail.sysu.edu.cn

**Keywords:** hand-foot-mouth disease, enterovirus 71, neutralizing antibodies, seroepidemiology

## Abstract

A hand-foot-mouth disease outbreak occurred in 2014 around Guangdong. The purpose of this study was investigating the status and susceptibility of infectious neutralizing antibodies to enterovirus 71 among children so as to provide scientific evidence for the population immunity level of hand-foot-mouth disease and prepare for enterovirus 71 vaccination implementation. Serum specimens were collected from children in communities from January 2014 to March 2015 in Guangzhou. A total of 197 serum samples from children 1–5 years old were collected for this cross-sectional study via non-probabilistic sampling from the database of Chinese National Science and Technique Major Project. Neutralization activity was measured via micro neutralization test in vitro. The positive rate of enterovirus 71 neutralizing antibodies was 59.4%, whereas the geometric mean titre was 1:12.7. A statistically significant difference in true positive rates was found between different age groups but not between different genders. Being the most susceptible population of hand–foot–mouth disease, children under 3 years of age are more likely to be infected with enterovirus 71, and the immunity of children increases with increasing age. Further cohort studies should be conducted, and measures for prevention and vaccination should be taken.

## 1. Introduction

According to the updated surveillance summary of World Health Organization [1], an outbreak of hand-foot-mouth disease (HFMD) was recorded in China in 2014. In 2015, 2,014,999 cases of HFMD were reported in China. Zhang [2] reported that the incidence of HFMD in Guangdong increased, peaking twice each year from 2009 to 2012 (incidence range was from 9.75 × 10^−5^ to 32.08 × 10^−5^). Enterovirus 71 (EV71, species *Enterovirus A*, genus *Enterovirus*, family *Picornaviridae*, order *Picornavirales*) is a major causative agent of HFMD. Jin [3] conducted an etiological study and found that 51% of HFMD cases in China in 2011 were caused by EV71. EV71 infection causes fever, skin eruptions on hands and feet, as well as vesicles in the mouth. Meningitis, encephalomyelitis, and neurogenic pulmonary oedema [4] may be involved in rare cases, which may cause serious sequelae or death [5]. EV71 seroepidemiological studies were conducted in Germany [6], Brazil [7], Singapore [8], Taiwan [9,10], and several cities in mainland China, such as Shanghai, Shenzhen [11], Henan [12], and Handan. Guangzhou, the capital of Guangdong, presents high population mobility in Southern China; Guangzhou residents, especially children, may be highly susceptible to EV71. Hence, few seroepidemiological studies focused on this region during the 2014 outbreak. Estimation of the seroprevalence of EV71 neutralizing antibodies and the level of herd immunity is important to provide the supporting principle for HFMD prevention and control strategies.

An individual who is susceptible to EV71 can be infected by asymptomatic infection. Sun [13] reported a high asymptomatic infection rate of EV71 (24%) during the epidemic season in Wenzhou in 2012. Given that HFMD is an acute self-limited infectious disease, humans can produce neutralizing antibodies by inducing immune responses, either with symptomatic infection or asymptomatic infection. EV71 neutralizing antibodies can be detected 1 or 2 weeks after infection and may persist for at least a year [14]. Xu [15] conducted a seroepidemiological research on 254 children in Henan between 2010 and 2012, and found a positive correlation (correlation coefficient *r* = 0.80) between the positive rate of EV71 neutralizing antibodies and morbidity within the same year. The status of EV71 neutralizing antibodies should be explored to determine HFMD morbidity and population immunity. By contrast, the seroepidemiological study of Rong [16] in Guangzhou revealed significant differences in seropositivity between the younger age group and the eldest group. The samples were obtained not only from children but also from adults aged 24–35 years. Therefore, investigation of the positive rate of EV71 neutralizing antibodies and the susceptibility to EV71 among children aged 1–5 years in Guangzhou is essential to provide the seroepidemiological information necessary for region disease control implementation and EV71 vaccination.

## 2. Materials and Methods

All subjects provided their signed informed consent to inclusion prior to participation in the study. The study was conducted in accordance with the 1964 Declaration of Helsinki, and the protocol was approved by the Ethics Committee of Institutional Review Board of School of Public Health, Sun Yat-sen University. The ethical code is L2016[021]. Our study was conducted as a cross-sectional study via non-probabilistic sampling. We calculated the sample size as 119 with an estimated neutralizing antibody positive rate (abbreviation as “p”) of 59%, significance level of 0.05, and permissible error of 0.15 “p”. Considering the small sample number for each age group, we collected 197 samples between 2014 and 2015. All serum specimens and demographic characteristics of children in Guangzhou were collected from the blood sample database set up by Chinese National Science and Technique Major Project (2012ZX10004912). Child participants were divided into four groups: under 2 years old, between 2 and 3 years old, between 3 and 4 years old, and between 4 and 5 years old. As positive control, the EV71 positive serum was collected from child patients identified by Yuebei Hospital in Shaoguan City. Human rhabdomyoma (RD) cells were provided by Guangdong Centre for Diseases Control (CDC) and used for virus sub-cultivation and antibody neutralization test. EV71 (virus strain number 2014XN37281) was collected from HFMD cases, isolated, cultured, and sequencing identified by Guangzhou CDC.

Virus titration was conducted via endpoint dilution assay. This assay was selected because of its higher sensitivity and cost-effectiveness compared with plaque assay [17,18]. Virus liquid was sequentially diluted by minimum essential medium (MEM) in gradient dilutions from 10^−1^ to 10^−10^ and then cultured in 96-well culture plates (8 × 12 wells). Eight wells per vertical row in each culture plate were for one dilution level of virus liquid inoculated (50 µL was added to each well). Each culture plate included one cultivating liquid group and one cell suspension group as controls. To prevent the infection from being trailed from non-neutralized virus wells to the cell culture suspension, all tips were changed for each new row. A 50% Tissue Culture Infective Dose (TCID_50_) was generated to measure the EV71 antibody titre, and mathematically calculated using the Reed–Muench method [19]. Observation and recording lasted for 3 days.

EV71 neutralizing antibody titre was determined by the absence of cytopathic effects (CPE) in the micro neutralization test [20,21]. Serum samples were diluted at a sample-treatment-liquid ratio of 1:4 and processed under homogeneous vibration. After being placed at a constant temperature of 4 °C overnight, the samples were inactivated the next day at 56 °C for 30 min. Then, the samples were diluted sequentially twice from 2^−1^ to 2^−9^ and cultured in 96-well culture plates within two wells for one dilution level (50 µL per well). Then, diluted 100 TCID_50_ virus liquid (50 µL per well) was added. To prevent cross infection, all tips were changed for each new row. After homogeneous vibration, these culture plates were placed in a carbon dioxide incubator for 2 h neutralization. RD cell liquid was added (100 µL per well), and the plates were placed back into the carbon dioxide incubator at 35 °C. RD cells were used for virus subcultivation. Occurrence of CPE in RD cell culture indicates the presence of virus activities. When EV71 neutralizing antibody is present in RD cell culture, the infectivity of EV71 is reduced, leading to the absence of CPE in the cell culture. Therefore, the absence of CPE proved the positive result of EV71 neutralizing antibodies. CPE of these 96-well culture plates was observed under an inverted microscope daily for 3 days. The serum control group, virus control group, and blank control group were added simultaneously.

Standard prediction was performed after the micro neutralization test. The multiplicative inverse of the highest dilution level under CPE absence was the determined point of neutralizing antibody titre. The antibody titre value determined the positive or negative results of EV71 neutralizing antibodies. If the neutralizing antibody titre values were less than or equal to 1:4, 1:4 was recorded as negative. The value was classified as positive if the neutralizing antibody titre values were greater than or equal to 1:8. All neutralizing antibody titre values that were greater than 1:32 were recorded as ≥1:32. Calculations of the geometric mean titre (GMT) of EV71 neutralizing antibodies and its 95% confidence interval (95% CI) were generated.

### Statistical Analysis

Statistical analysis of data was performed by SPSS 19.0 (IBM SPSS Statistics, Armonk, NY, USA). Chi-square test was used to compare the positive rates of EV71 neutralizing antibodies within different ages, genders, and years. Analysis of variance (ANOVA) was used to compare the logarithm values of neutralizing antibodies GMT within different ages, genders, and years. Cochran-Armitage Trend Test was performed to verify the trend of positive rates along with age. The significance level was set to 5% (α = 0.05). Pairwise comparison of the neutralizing antibody GMT between two age groups was processed in Fisher’s least significant difference (LSD) with significant level correction.

## 3. Results

### 3.1. Demographic Information

In total, 197 children aged 1–5 years participated in this study. The participants comprised 112 males and 85 females (male-to-female ratio was 1.3:1). Groups of children aged <2, 2–3, 3–4, and 4–5 years included 17, 60, 60, and 60 samples, respectively.

### 3.2. Presence or Absence of CPE

**Figure 1:**
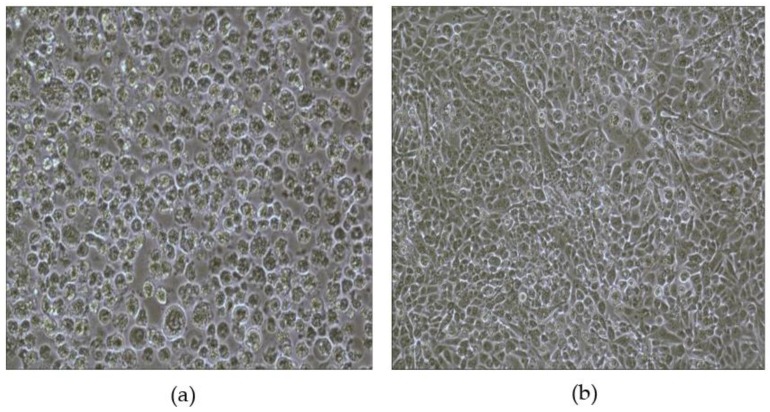
(**a**) RD cells with CPE; (**b**) normal RD cells; absence of CPE testified the positive result of EV71 neutralizing antibodies; multiplicative inverse of the highest dilution level without the presence of CPE was the determined point of EV71 neutralizing antibody titre.

RD cells can support the replications of EV71; therefore, CPE occurred in the RD cell culture as shown in Figure 1a, indicating the presence of EV71 activity. The absence of CPE shown in Figure 1b indicates a positive EV71 neutralizing antibodies result.

### 3.3. Results between Different Age Groups

**Figure 2:**
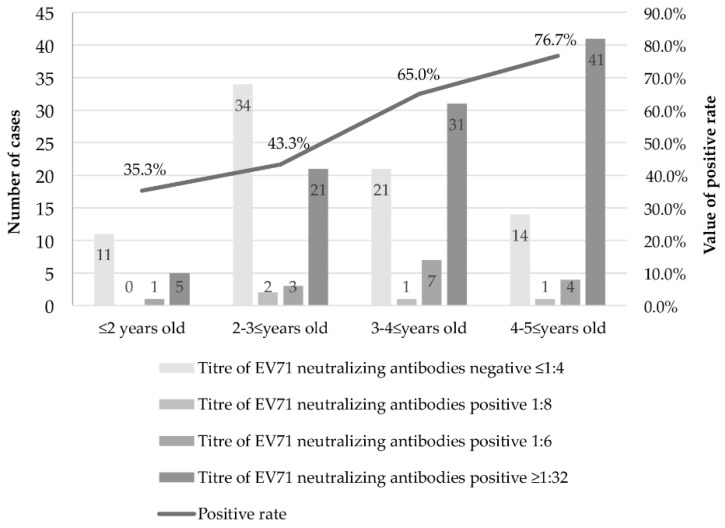
Antibody titre and positive rates of EV71 neutralizing antibodies in different age groups of children. Negative results were determined by titres of antibodies (≤1:4), and positive results were determined by titres of antibodies (1:8, 1:16, and ≥1:32). All neutralizing antibody titre values that exceed 1:32 were recorded as ≥1:32. Positive rates were generated from all positive results.

**Figure 3:**
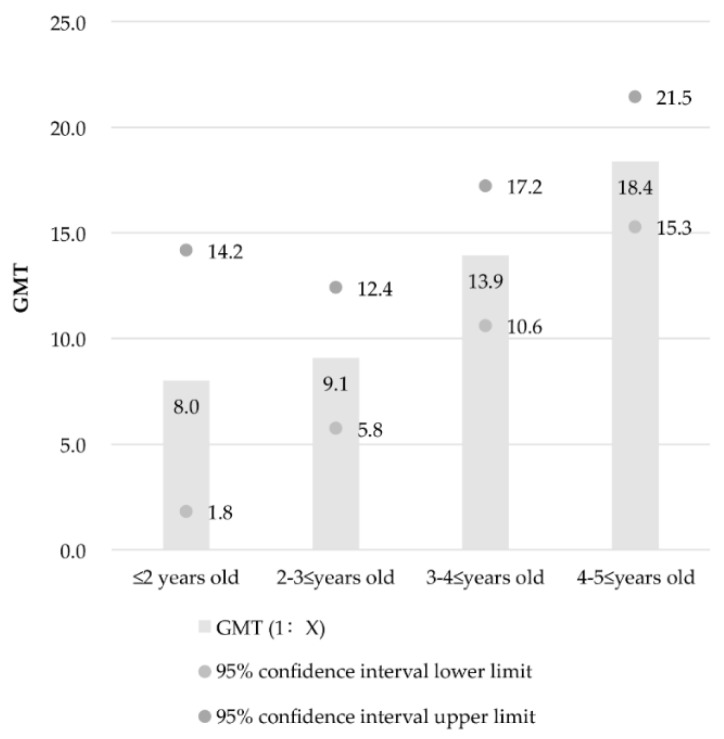
Geometric mean titre (GMT) and GMT 95% confidence intervals of EV71 neutralizing antibodies in different age groups of children.

As shown in Figure 2, 117 specimens tested positive among the 197 serum specimens of children (positive rate was 59.4%). The GMT of EV71 neutralizing antibody was 1:12.7 (95% CI: 1:10.8, 1:14.6). The positive rates of EV71 neutralizing antibodies in the groups of children aged <2, 2–3, 3–4, and 4–5 years were 35.3% (6/17), 43.3% (26/60), 65.0% (39/60), and 76.7% (46/60), respectively. The positive rates significantly increased with increasing age (*z* = 3.361, *p* < 0.001). Statistically significant differences in positive rates were observed among the four groups (Pearson χ^2^ = 18.715, *p* < 0.05). The total GMT value of EV71 neutralizing antibodies was 1:12.7 (95% CI: 1:10.8, 1:14.6). Furthermore, the GMT values were 1:8.0 (95% CI: 1:1.8, 1:14.2), 1:9.1 (95% CI: 1:5.8, 1:12.4), 1:13.9 (95% CI: 1:10.6, 1:17.2), and 1:18.4 (95% CI: 1:15.3, 1:21.5) in the four groups (Figure 3). More neutralizing antibodies were detected in the serum of the elder-age group than in the sera of the other age groups of children.

Statistically significant differences in the logarithm values of GMT were observed among the four groups (F = 7.092, *p* < 0.05). However, statistically significant differences in GMT only were found between the groups of children aged <2 and 4–5 years (*p* = 0.002), as well as between the groups of children aged 2–3 and 4–5 years (*p* = 0.000077).

### 3.4. Results between Different Genders

**Figure 4:**
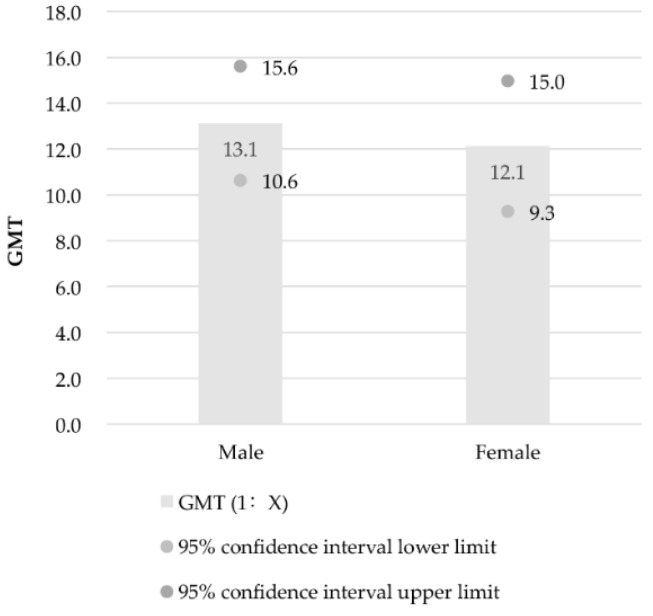
GMT and GMT 95% confidence intervals of EV71 neutralizing antibodies in different genders of children.

The positive rates of EV71 neutralizing antibodies were 59.8% for boys and 58.8% for girls. Among the boys’ titre values, 40.2% (45 samples out of 112) were less than 1:4, 8.0% (9/112) were 1:16, and 51.8% (58/112) were equal or greater than 1:32. Among the girls’ titre values, 41.2% (35/85) were less than 1:4, 4.2% (4/85) were 1:8, 7.1% (6/85) were 1:16, and 47.1% (40/85) were equal or greater than 1:32. No statistically significant difference in positive rates of EV71 neutralizing antibodies were observed between different genders (*p* = 0.888). The GMT values of EV71 neutralizing antibodies were 1:13.1 (95% CI: 1:10.6, 1:15.6) for boys and 1:12.1 (95% CI: 1:9.3, 1:15.0; Figure 4) for girls. No statistically significant difference in GMT was found between different genders (*p* = 0.579).

## 4. Discussion

Enterovirus 71 is the major pathogen that causes HFMD, especially among children. In Shenzhen, China, where the Pearl River delta is located along with Guangzhou, the positive rate of EV 71 Immunoglobulin G (IgG) antibodies was 45.64% in 2012 among children below 5 years of age [22]. According to Kuang [23], this value was 30.83% in Guangzhou in 2010. These findings show that the positive rate of antibodies in Guangzhou was higher in this study (59.4%) than before, indicating that the immunity of children increased during the past 5 years. Zeng [24] observed an inverse correlation between specific age and EV71-infected HFMD cases in Shanghai. Therefore, individual susceptibility to EV71 could be determined by the level of EV71 neutralizing antibodies. In the present study, statistically significant differences in positive rates, as well as in the logarithm values of GMT, were observed among the four age groups. Therefore, we can conclude that during 2014 and 2015, different groups of children under 5 years of age in Guangzhou presented various levels of susceptibility to EV71. Moreover, the positive rate for elder children was higher than that for younger children, implicating that children aged less than 3 years were the most susceptible population. Zhou [11] conducted a seroepidemiological study of EV71 in 2007 in Shenzhen and found that the positive rate of antibodies (30.8%) in the elder group (2–5 years old) was higher than that in the younger groups (1–2 years old and <1 years old, 19.3% and 19.0% respectively). The results of the present study agree with those found by Zhou. Given that children under this age are either under home care (under 2 years old) or going to nursery school (between 2 and 3 years old), preventive measures at home and in school should be reinforced, including avoidance of direct contact with infected persons and proper hand-washing procedures.

Focusing on the details for each group, we found the lowest positive rate of EV71 neutralizing antibodies in children under 2 years old (35.3%), and the GMT of this group was 1:8.0. Similar results were obtained by Li [25] in Guangdong, China and by Linsuwanon [26] in Thailand, indicating that children under 2 years of age presented the lowest seropositive rate of EV71 neutralizing antibodies. Children between 4 and 5 years old in this study showed the highest positive rate (76.7%) among all the groups, presenting more than two times the positive rate for children under 2 years of age. Younger children with incomplete immune system may show more than two times the probability of being secondary infected. In Jin’s [3] HFMD epidemic features study in 2011, morbidity rapidly increased with age under 1 year of age and reached the peak of morbidity. Among children older than 1 year, morbidity decreased rapidly after the peak but remained on a higher level with increasing age. The high morbidity of HFMD at this age is due to the fluctuation in the amount of maternal antibodies, as well as the unstable, incompletely developed immune system function of children. In Ooi’s [9] study from Singapore, 44.0% of pregnant mothers possessed EV71 neutralizing antibodies, which waned rapidly. After a month, none of the new-born babies who were tested possessed maternal antibodies to EV71. With low immunity level and rapidly decreasing number of maternal antibodies during child growth, the risk of infection would be higher if the children did not possess the EV71 neutralizing antibody. Younger children are less aware of HFMD risk and good hygienic preventive actions, and also lack supervision because of the insufficient numbers of nurses or teachers in nursery schools and kindergartens. These children are more easily infected by EV71 via respiratory droplets from close contact. Meanwhile, children aged beyond 4 years show decreasing morbidity of HFMD, which may be associated with recessive infection or higher positive rate of EV71 neutralizing antibodies. Raising public awareness of HFMD in children under 3 years of age, broadcasting HFMD general information and its prevention measures, improving the situation of health care in kindergartens and nursery schools, and implementing vaccines against EV71 are needed. Proper hand hygienic preventions, especially for children under 3 years of age, should be supervised by the guardians and tutors. Currently in Mainland China, inactivated vaccines against EV71 passed phase III clinical trial [27,28], and these inactivated vaccines as Class 1 preventive biological products received the approval of production registration from China Food and Drug Administration (CFDA) in 2015. Children under 3 years of age should be primarily considered as the targeted population for vaccination. The seroprevalence of EV71 neutralizing antibodies based on age groups would be useful to establish immunization protocols.

The positive rates of EV71 neutralizing antibodies for boys and girls were 59.8% and 58.8%, showing no statistical significance between genders. In a German study on 696 individuals, Rabenau [29] found that the positive rate of EV71 neutralizing antibodies in males is 41.3%, which is higher than that of females (44.4%), but the difference is not statistically significant. Similarly, Horwood [30] found that the seroepidemiology of EV71 neutralizing antibodies relative to genders in Cambodia does not show a significant difference (89.8% for females and 87.7% for males, *p* = 0.18). Consistent with the results of these studies, the transmission of EV71 can be inferred to be nonselective toward gender in the Guangzhou population. By contrast, no significant differences in positive rate or GMT were found among children in Guangzhou between 2014 and 2015. This outcome differed from the findings of Li [25] in 2013 that the seropositivity rate of EV71 neutralizing antibodies in Guangdong significantly increased after the 2010 epidemic. In the comparison of the two findings from the same region, the Guangzhou 2014 HFMD outbreak did not affect the antibody immunity level among children. However, these results could also be due to the closeness of the collection years 2014 and 2015 for observing a significant difference. The surveillance of EV71 should continue even after the HFMD outbreak, and inactivated vaccines against EV71 are emergently needed, particularly for children (both boys and girls) under 3 years of age, after the 2014 HFMD outbreak to improve the immunity of the susceptible population.

The limitation of our study is the different number of samples for each age group. The sample size of children under 2 years of age was markedly smaller than those of the three other groups. Chang [31] indicated that the highest mortality rate (15.6/10^5^) can be found in children between 6 and 11 months old. Therefore, further seroepidemiological study on EV71 should be continued, including sample collection from under children below 1 year of age. As an important factor of the detection prevalence validation of EV71 neutralizing antibodies, the precise duration of EV71 neutralizing antibodies should be investigated. Given that the susceptible population involves children under 3 years old, specific medical treatments and vaccination to EV71 for children should be under the national and regional implementations.

## 5. Conclusions

Children under 3 years of age are highly susceptible to HFMD. During 2014 and 2015, the positive rate of EV71 neutralizing antibodies among 197 serum specimens from children aged 1–5 was 59.4%, whereas the GMT was 1:12.7. Statistically significant differences in positive rates were observed between different age groups but not between different genders. Given the HFMD transmission routes and the characteristics of susceptible population, prevention and control measures should be taken. Further epidemiological studies and vaccination to EV71 should be continued under the national and regional protocols of implementation.
